# Comment on ‘comparison of overall survival and perioperative outcomes of laparoscopic pancreaticoduodenectomy and open pancreaticoduodenectomy for pancreatic ductal adenocarcinoma: a systematic review and meta-analysis’

**DOI:** 10.1186/s12885-020-06830-4

**Published:** 2020-04-16

**Authors:** Rui Sun, Yifan Zhang, Zhe Su

**Affiliations:** 1 Department of Pediatric Surgery, Capital Institute of Pediatric, No. 2 Yabao Road, Beijing, 100020 China; 2 Chinese Academy of Medical Sciences and Peking Union Medical College, Beijing, China

## Abstract

**Background:**

To discuss some inaccurate parts of a published systematic review and meta-analysis which was about comparison of laparoscopic pancreaticoduodenectomy and open pancreaticoduodenectomy for pancreatic ductal adenocarcinoma in overall survival and perioperative outcomes.

**Methods:**

Not applicable.

**Results:**

The study enrolled overlapping patient cohorts and patients with a variety of disease histologies, not specific for pancreatic ductal adenocarcinoma. They also reported different conclusion for R0 resection of 2 approaches in result and discussion part. Some data should be revised through statistical method, which could be polled to analysis.

**Conclusion:**

The conclusion of this published systematic review and meta-analysis was not objective; therefor, the study may mislead readers, especially for those who just read the abstract of this study or not analysis this article in detail.

## Main text

A recently study by Jiang et al. [1] compared overall survival and perioperative outcomes of laparoscopic pancreaticoduodenectomy (LPD) and open pancreaticoduodenectomy (OPD) for pancreatic ductal adenocarcinoma: a systematic review and meta-analysis, which was published in August. The author claimed that they evaluate clinical efficacy of LPD and OPD for the treatment of Pancreatic ductal adenocarcinoma (PDAC). However, we believe their study has not provided an objective conclusion regarding the oncologic and perioperative outcomes of LPD and OPD in setting of PDAC for the following reasons.

First, 8 studies were included in their meta-analysis, whereas studies of Sharpe et al. [2] and Kantor et al. [3] had overlapping patient cohorts. Their data was both provided by National Cancer Data Base. Sharpe et al. identified all patients 18 years and older diagnosed with PDAC who underwent an LPD or OPD between January 2010 and December 2011, while Kantor et al. analyzed data from 2010 to 2013. Therefore, those describing the smaller-scale studies should be excluded in the meta-analysis.

Second, the objective of their study was to confirm efficacy of LPD in patients with PDAC. However, the studies of Speicher et al. [4] and Chen et al. [5] involved patients with a variety of disease histologies, not specific for PDAC. Speicher et al. enrolled 140 patients in total (25 in LPD, 84 in OPD), only including 104 cancer cases without clearly histopathological types reported. Chen et al. involved identified 102 patients having undergone pancreaticoduodenectomy with a pathologically confirmed diagnosis of periampullary tumors, bile duct carcinoma, intra-ductal papillary mucinous neoplasms and pancreatic head cancer between January 2013 and May 2017. We believe these two studies should not be included in the meta-analysis to provide the conclusion.

Third, they demonstrated that LPD resulted in a higher rate of R0 resection compared with OPD. The resulted was showed in Fig. 3 in their study (OR: 1.16, 95% CI 0.85–1.57, *p* = 0.36). Since the *P* value was greater than 0.05 the result indicated there was no significant difference between two approaches in R0 resection. Therefore, we believe the conclusion of LPD in R0 resection was incorrect.

In addition, according to the methods of Wan et al. [6], medians with ranges could be converted into means with standard deviations, which should be calculated to pooled the results in the analysis, including R0 resection, estimated blood loss and hospital stay. We suggested the outcomes may be different if they pooled all available data.

Reading of the abstract or failure to analyze the paper in detail may provide the reader with the impression that LPD is equivalent to OPD with respect to overall survival and results in better perioperative clinical outcomes for patients with PDAC whereas we believe this is not the case. The paper does not demonstrate convincingly that LPD introduces equivalent or even improved outcomes compared to OPD, especially for PDAC.

## Data Availability

Not applicable.

## Abbreviations

LPD: Laparoscopic pancreaticoduodenectomy;
OPD: Open pancreaticoduodenectomy;
PDAC: Pancreatic ductal adenocarcinoma;
OR: Odds ratio
